# Dependence to legally prescribed opioid analgesics in a university hospital in Medellin-Colombia: an observational study

**DOI:** 10.1186/s40360-016-0087-4

**Published:** 2016-09-14

**Authors:** Maria G. Garcia-Orjuela, Lineth Alarcon-Franco, Juan C. Sanchez-Fernandez, Yuli Agudelo, Andres F. Zuluaga

**Affiliations:** 1 Departamento de Farmacología y Toxicología, CIEMTO: Centro de Información y Estudio de Medicamentos y Tóxicos, Facultad de Medicina, Universidad de Antioquia, Carrera 51D No. 62-42, Medellín, Colombia; 2 Hospital Universitario San Vicente Fundación, Calle 64 No. 51 D–154, Medellín, Colombia; 3 GRIPE: Grupo Investigador de Problemas en Enfermedades Infecciosas, Facultad de Medicina, Universidad de Antioquia, Calle 70 No. 52-21, Medellín, Colombia

**Keywords:** Analgesics, Opioid-related disorders, Tramadol, Pain

## Abstract

**Background:**

In some countries the misuse and diversion of prescribed opioid analgesic is increasing considerably, but there is no official data regarding the situation in Colombia. The aim of this study was to identify all dependent to opioid analgesics legally prescribed patients that were treated in a University Hospital in Medellin, Colombia during 4 years and to characterize this population.

**Methods:**

Observational study in a University Hospital in Medellin, Colombia, searching for patients with ICD-10 codes related with opioid related disorders, adverse events or pain and treated between January 2011 and December 2014.

**Results:**

Sixty patients with opioid dependence according to DSM-IV criteria were found from 3332 clinical charts reviewed. The median age was 43 years. Although all patients met the DSM-IV criteria, 33 % of patients were wrongly diagnosed by other ICD-10 codes. Almost all patient (88 %) initiated opioids after medical prescription although the adherence to pain scale was low (25 %). The median time of consumption was 48 months. Tramadol was the opioid more frequently used by patients, followed by morphine and oxycodone. A statistically significant higher consumption of other psychotropic substances was observed in male than female (*P* = 0.005 by Fisher’s test). After be diagnosed, 55 % of patients gone a methadone-based replacement therapy.

**Conclusion:**

Legally prescribed opioid dependence was belatedly diagnosed in 60 patients in a University hospital, after prolonged use of drugs to treat chronic pain and with low adherence to pain scale or guidelines. This is the first report in Colombia.

## Background

The opioid analgesics are essential medications in the management of acute and chronic pain [1]. Although the effectiveness of opioid therapy to improve chronic pain is questionable [2], the successful pain relief may require high doses and longtime usage of opioids, increasing the risk of abuse and dependence disorders induced by these drugs [3].

Abuse of legally prescribed opioids could be potentiated because these drugs are broadly available, are manufactured by well-recognized pharmaceutical companies, and there is a perception of safeness during the use of opioids by the people, minimizing its addictive potential [4]. The medical dilemma is simple, physicians need opioid drugs as treatment but there is an unequivocal risk of abuse and dependence that should be considered and minimized.

In some countries, the abuse and diversion of prescribed opioid analgesic is growing since two decades ago [5]. In United States, 25 million people consumed legal opioids for nonmedical uses between 2001 and 2011 [6]. More than 10 % of the admissions for treatment in opioid abuse in this country and 16,651 deaths were attributed to prescription opioid drugs [6]. Currently, in Australia and New Zealand the opioid abuse and dependence is at least three fold more common than similar disorders induced by heroin [1, 4, 6].

However, in Colombia there are no official statistics of the misuse of prescription opioid medications, and apparently, in contrast to other countries it is not considered as a real problem by government agencies. This lack of data contradicts the increasing clinical observations of new patients treated by dependence to legally prescribed opioids in our country. The aim of this study was to identify all patients with opioid analgesic dependence that were treated in a University Hospital in Medellin, Colombia during 4 years and to characterize this population.

## Methods

### Study

Retrospective observational study reviewing the clinical chart of all patients treated for dependency to legally prescribed opioids in the University Hospital (Hospital Universitario San Vicente Fundacion) in Medellin, Colombia between January 2011 and December 2014.

### Sample size and population

As there were neither reference studies nor government statistics, a non probabilistic sampling was used. To identify the cases, a systematical review of all registries of out- and inpatients reported by the University Hospital was done, looking for the following ICD-10 codes potentially related with dependence to legally prescribed opioids: (a) opioid related disorders: opioid abuse, (b) opioid related disorders: opioid dependence, (c) opioid related disorders: with withdrawal, (d) opioid related disorders: with other opioid-induced disorder, (e) opioid related disorders, unspecified, (f) acute pain, (g) chronic intractable pain, (h) other chronic pain, (i) pain, unspecified, (j) poisoning: other opioids, and (k) opioids and related analgesics: adverse effects.

All the clinical records of the adult patients (aged 18 or older) associated with at least one of the ICD-10 codes specified above and treated in University Hospital during the period of the study, were reviewed by some of the authors (all physicians) to select the cases which fulfilled the DSM-IV diagnostic criteria for opioid dependence, which includes three or more of the following, occurring at any time in the same 12-month period: (a) tolerance, (b) withdrawal, (c) the substance is often taken in larger amounts or over a longer period than was intended, (d) there is a persistent desire or unsuccessful efforts to cut down or control substance use, (e) a great deal of time is spent in activities necessary to obtain the substance or recover from its effects, (f) important social, occupational, or recreational activities are given up or reduced because of substance use, (g) the substance use is continued despite knowledge of having a persistent or recurrent physical or psychological problem that is likely to have been caused or exacerbated by the substance [7].

The exclusion criteria included illegal opioids dependence and mixed disorders (i.e., consumption of illegal and legal prescribed opioids).

### Data collection

For the data collection a questionnaire was designed according to the study variables in Google Forms. Three of the trial physicians reviewed all the clinical records with the pre-established ICD-10 codes, and fulfilled a questionnaire for every patient.

### Statistical analysis

A simple descriptive analysis for all variables was performed. Discrete data were summarized as percentage while continuos ones were presented as median with range from minimum to maximum. To determine the relationship between two categorical variables, the data of nominal variables as demographic variables (i.e., marital status, origin, education level, etc) or consumption patterns (i.e., number of patients consuming opioid for diversion, self-medication, etc), were compared between genders by Chi-square independence test or Fisher’s exact test (when the expected number of subjects in a single cell was less than 5). The information was processed with the statistical package Instat® (Graphpad, USA).

## Results

A total of 3332 records were obtained from the University Hospital database, both inpatient and outpatient, since January 2011 to December 2014. A total of 60 patients fulfilled the DSM-IV criteria for opioid dependence.

The patients and their main demographics characteristics distributed by gender are shown in Table 1. The population studied was equally distributed between male and female with 30 patients in each case, and there were no statistically significant differences comparing all demographic variables by gender (*P*-value >0.11 by Fisher’s exact test). The median age was 43 years, ranging from 18 to 87 years old. In general, the most frequent kind of patient included was single, widower or divorced, from the metropolitan area, employed with some level of education.

**Table 1:** Demographic characteristics of the population by gender

Variable	Options	Male *N* = 30 (%)	Female *N* = 30 (%)	Total *N* = 60 (%)	*P*-value
Age	18–60 years old	30 (100)	26 (87)	56 (93)	0.11^a^
	>60 years old	0 (0)	4 (13)	4 (7)	
Marital Status	Single, widowers or divorced	14 (47)	15 (50)	29 (48)	0.89^b^
	Married & cohabiting	13 (43)	13 (43)	26 (44)	
	No data	3 (10)	2 (7)	5 (8)	
Origin	Metropolitan Area	18 (60)	15 (50)	33 (55)	0.73^b^
	Outside The Metropolitan Area	6 (20)	7 (23)	13 (22)	
	No data	6 (20)	8 (27)	14 (23)	
Occupation	Employee	19 (63)	23 (77)	42 (70)	0.50^b^
	Unemployed	7 (23)	5 (17)	12 (20)	
	No data	4 (14)	2 (6)	6 (10)	
Education Level	Undergraduate	15 (50)	19 (63)	34 (57)	0.35^b^
	Postgraduate	7 (23)	3 (10)	10 (16)	
	No data	8 (27)	8 (27)	16 (27)	

^a^ *P*-value by Fisher’s exact test

^b^ *P*-value by Chi-square test

Table 2 summaries the clinical characteristics related with dependence to legally opioids in the 60 patients studied. It should be noted that only two thirds of patients involved were diagnosed with opioid related disorders, meaning that up to 33 % of patients with DSM-IV criteria to dependence to opioids were undiagnosed or clinically treated by other reasons without suspicious of this disorder. Additionally, almost all of patients (88 %) initiated on opioids by a medical prescription to treat any type of pain, although the description of the use of a pain scale was minimum (25 %) in our population. In this context, could be not surprising that the perpetuation of the opioids misuse was related with self-medication of these drugs mainly justified by an apparent pain. Only 4 of 60 patients (7 %) had an oncologic diagnosis.

**Table 2:** Clinical description of the characteristic related with dependence to legally opioids observed in the 60 patients studied

Description	Yes (%)	No (%)	No data (%)
Number of patients with opioid related disorders according to ICD-10 codes (F11.1, F11.2, F11.3, F11.8 and F11.9)	40 (67)	20 (33)	0 (0)
Number of patients involved in the study that started the consumption of opioids by legal prescription to treat any pain	53 (88)	4 (7)	3 (5)
Number of patients with description in the clinical chart of the pain rating scale use previous to the first prescription of opioid	15 (25)	16 (27)	29 (48)
Number of patients suffering clinical complications (i.e., psychosis) related to opioid consumption	8 (13)	47 (78)	5 (8)
Number of patients with dependence to legal opioids perpetuated by self-medication	54 (90)	2 (3)	4 (7)
Number of patients with dependence to legal opioids perpetuated also by diversion consumption for nonmedical use	9 (15)	32 (53)	19 (32)
Number of patients with description in the clinical chart of simultaneous use of psychotropic substance(s) as tobacco, alcohol and others	19 (32)	27 (45)	14 (23)

**Fig. 1:**
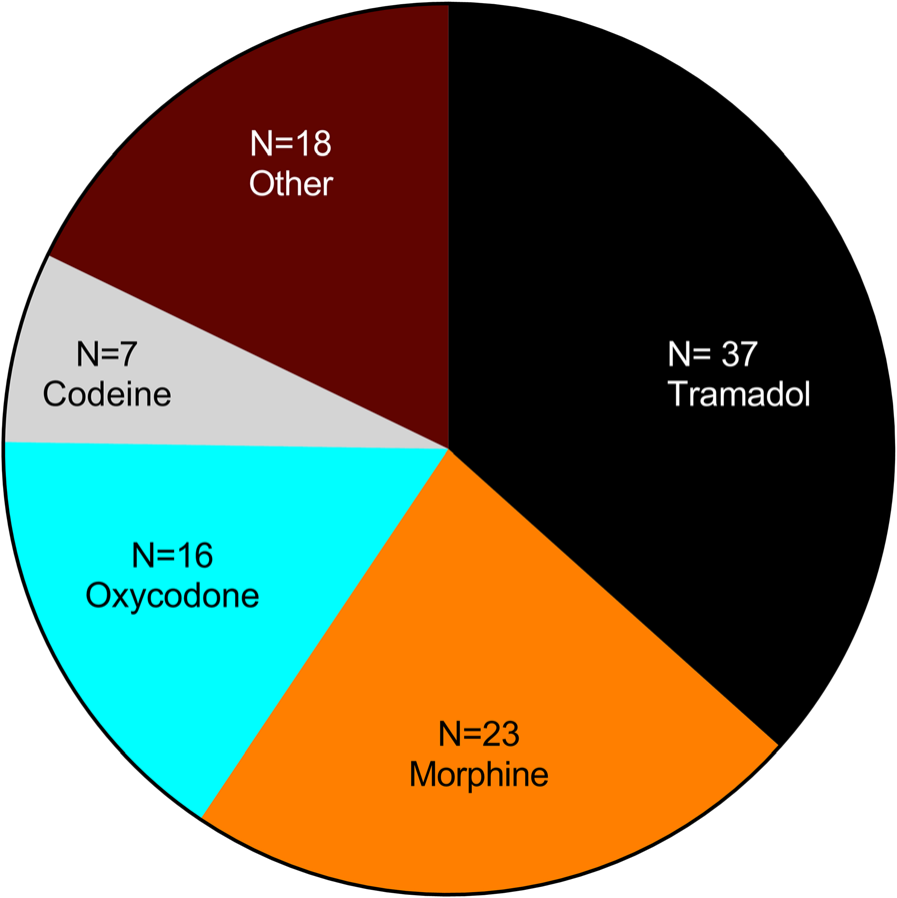
Frequencies of use by type of opioid in patients. The total number is higher than 60, because all patients could abuse of opioids using as single drug or mixing it

Regarding the characteristic of the consumption, the more frequently used opioid by patients involved was tramadol, followed in order by morphine, oxycodone and others (Fig. 1). One opioid was used by 23 subjects as single drug related to the dependence, the remaining persons received or rotate by multiple opioids. The more common rotation of drugs was using morphine and tramadol (6 of 37 patients, 16 %). The median time of consumption was 48 months, ranging from 1 to 240.

The consumption of other psychotropic substances associated with opioid dependence was found in 15 (25 %) male patients and 4 (7 %) female patients, with statistical significance (*P* = 0.005); in general, 37 % consumed tobacco, 21 % alcohol, and 16 % other psychotropic substances not specified (Table 3). It should bear in mind that the data from 11 (18 %) patients was missing.

**Table 3:** Comparison of the pattern of consumption in the 60 patients by gender

Variable	Description	Male *N* = 30 (%)	Female *N* = 30 (%)	Total *N* = 60 (%)	*P*-value by Fisher’s exact test
Diversional use	Number of patients consuming opioid for diversion	7 (23)	2 (7)	9 (15)	0.15
Combination of opioids	Number of patients with consumption of more than one opioid	18 (60)	19 (63)	37 (62)	1.00
Combination with other substances	Number of patients using other psychotropic substances	15 (50)	4 (13)	19 (32)	0.005
Prolonged consumption	Number of patients with opioid consumption for more than 12 months	23 (77)	23 (77)	46 (77)	1.00
Therapeutic management	Number of patients receiving any treatment	28 (93)	29 (97)	57 (95)	1.00
Methadone-based replacement therapy	Number of patients treated with methadone as replacement therapy	19 (63)	14 (47)	32 (55)	0.30

Among the patients with opioid misuse, 32 (55 %) had been started on replacement therapy with methadone after months or years of diagnosis of opioid dependence by DSM-IV criteria, and 24 (40 %) patients had undergone other unspecified treatment. In 1 case (2 %) no treatment was started, and there was no any treatment information for other two patients (3 %).

Finally, DSM-IV criteria more frequently found were that the substance was often taken in larger amounts or over a longer period in 59 patients (98 %), followed by tolerance in 57 cases (95 %), withdrawal in 57 users (95 %), an unsuccessful persistent desire to cut down the misuse in 54 subjects (90 %), and a great deal of time is spent in activities necessary to obtain the substance or recover from its effects just in 30 patients (50 %), the remaining were present in less than 46 % of population studied.

## Discussion

The opioid analgesic dependence (OAD) is a well-recognized health problem in many countries [4], it is even considered an epidemic problem [8, 9]. In United States, the number of opioid overdose deaths are similar to number of deaths from car accidents [10]. Here, we described the first observational study performed in Colombia corroborating that OAD is also a real and problematic situation.

Although there is no official data, it has not scape our attention that our study performed in just one high specialized hospital in Medellin, Colombia, were more than enough to detect 60 patients during 4 years, that is approximately 15 cases per year. This finding supports the importance to perform a national surveillance including multiple medical centers to identify the prevalence of this disease.

It has been described that most of the patients with OAD initially obtained the drug misused from a physician, but then the patient abuse of this formulation, whether looking for an euphoric effect or for avoiding the withdrawal symptoms [11]. In our study, we described a similar behavior, that is 88 % of patients started on opioids by a medical prescription to treat any type of pain with a low adherence to pain guidelines or scales, highlighting the utility of education to improve the adherence to national guidelines, better methods for diagnosis and monitoring, and stronger laws in Colombia that help control this problem with high risk to increase in short term.

Demographic characteristics of our population were closer to other studies [12–14], that is OAD are more common in median age patients, without difference by gender, and being initially prescribed for non-cancer pain management. This situation is probably showing the increasing use of opioids for acute and chronic non-cancer pain and the lack of a stepped pain treatment and the fail to follow the current recommendations as well [15].

In other countries, hydrocodone is currently topping all prescriptions of prescription opioids [8]. Unlike those statistics, tramadol was the initial and most frequent prescribed and misused opioid. This result is similar to the reported by Zhang et al., describing that the long period of use and/or high doses of tramadol may be one important risk factor of abuse even for those with no prior drug abuse [16]. Tramadol is a weak mu opioid receptor agonist and also possesses catecholamine reuptake inhibitor property [17]. It has been postulated that M1 metabolite of the drug mediates these actions. Mu receptors are involved in the euphoric symptoms related with the acute use of opioids [18], but their role in the dependence and abuse is still controversial [19]. Recently, Enabah et. al. demonstrated a statistically significant higher frequency of alleles CC and CT at site 3435 (rs1045642) of the ABCB1 gene in patients with dependence [20]. It should be noted that the same single nucleotide polymorphism in ABCB1 genes was found in Colombian patients with addictive behavior and addicted to heroin and cocaine. Then, it seems viable that genetics may contribute to the high frequency of tramadol use in patients with OAD [21].

The misuse of the opioid medications, is a clear risk factor for substance use disorder. It has been found that the patients with both disorders usually report more pain and impairment and depression disorders as well [22]. In this study, it was found that 22 patients (37 %) had associated consumption of other psychotropic substances, being statistically significant more prominent on males than in women (*P* = 0.005).

In our study one of the most striking results was the opioid use median of 48 months. This is no a minor point, because the long term use of prescription opioids is one of the main risk factors to mortality [8].

Almost all patients (≥90 %) met only four DSM-IV diagnostic criteria for prescription opioid disorder, the remaining criteria were lesser frequent (≤50 %). Given this frequency, it could be proposed to divided the diagnostic criteria in major (common) and minor (uncommon). During the accomplishment of this trial the DSM-V was published proposing a severity scale related with the number of criteria observed in each patient.

As retrospective study, it has some limitations, as the lack of control of the use of additional pain medication in each patient, neither the dose of opioid used or administered prior or during the hospitalization.

Finally, this observational study can be considered the first evidence that OAD is also diagnosed in Colombia. More prospective and controlled researches, also as national surveillance are required on this topic and maybe the enactment of laws promoting new strategies as abuse-deterrent products.

## Conclusion

The prescription opioid disorder is a growing health problem not only worldwide but also in Colombia, where currently there are no official statistics of this problem. It is indispensable to start educating the health personnel on the correct use of prescription opioids, being careful in its indications, type of drug use, and time of treatment.

## Abbreviations

CIEMTO: Centro de Información y Estudio de Medicamentos y Tóxicos
GRIPE: Grupo Investigador de Problemas en Enfermedades Infecciosas
ICD-10: International Statistical Classification of Diseases and Related Health Problems - 10th revision
DSM-IV: Diagnostic and statistical manual of mental disorders, Fourth edition
OAD: Opioid analgesic dependence
DSM-V: Diagnostic and statistical manual of mental disorders, Fifth edition
